# Stimulating somatosensory psychophysics: a double-blind, sham-controlled study of the neurobiological mechanisms of tDCS

**DOI:** 10.3389/fncel.2015.00400

**Published:** 2015-10-07

**Authors:** Claire J. Hanley, Mark Tommerdahl, David J. McGonigle

**Affiliations:** ^1^Cardiff University Brain Research Imaging Centre, School of Psychology, Cardiff UniversityCardiff, UK; ^2^School of Biosciences, Cardiff UniversityCardiff, UK; ^3^Department of Biomedical Engineering, University of North Carolina at Chapel HillChapel Hill, NC, USA

**Keywords:** transcranial direct current stimulation, neuromodulation, vibrotactile adaptation, amplitude discrimination, somatosensory, GABA, NMDA, Bayesian statistics

## Abstract

The neuromodulation technique transcranial direct current stimulation (tDCS) is thought to produce its effects on behavior by altering cortical excitability. Although the mechanisms underlying the observed effects are thought to rely on the balance of excitatory and inhibitory neurotransmission, the physiological principles of the technique are not completely understood. In this study, we examine the influence of tDCS on vibrotactile adaptation, using a simple amplitude discrimination paradigm that has been shown to exhibit modifications in performance due to changes in inhibitory neurotransmission. Double-blind tDCS (Anodal/Sham) of 1 mA was delivered for 600 s to electrodes positioned in a somatosensory/contralateral orbit montage. Stimulation was applied as part of a pre/post design, between blocks of the behavioral tasks. In accordance with previous work, results obtained before the application of tDCS indicated that amplitude discrimination thresholds were significantly worsened during adaptation trials, compared to those achieved at baseline. However, tDCS failed to modify amplitude discrimination performance. Using a Bayesian approach, this finding was revealed to constitute substantial evidence for the null hypothesis. The failure of DC stimulation to alter vibrotactile adaptation thresholds is discussed in the context of several factors that may have confounded the induction of changes in cortical plasticity.

## Introduction

Transcranial direct current stimulation (tDCS) is a neuromodulation technique capable of producing alterations in human behavioral performance, which are thought to rely on region-specific, polarity based changes in cortical excitability (Wassermann and Grafman, 2005; Utz et al., 2010; Paulus, 2011; Krause et al., 2013). Since the advent of the method, a number of studies have attempted to further elucidate the proposed mechanisms by which these changes in behavior occur. For example, the application of tDCS has been shown to alter the usual response of voltage-gated ion channels responsible for maintaining resting membrane potential (as documented in a recent review; Funke, 2013). When a positive (anodal) current is delivered to the cortex it has been proposed to lead to a depolarization of underlying neurons and following administration of a negative (cathodal) current, a state of hyperpolarization is said to be induced. Although this explanation may be greatly over-simplified (de Berker et al., 2013; Rahman et al., 2013), the induction of spatially specific depolarization and hyperpolarization have been supported by both animal (Creutzfeldt et al., 1962; Bindman et al., 1964; Purpura and McMurtry, 1965) and human studies (Nitsche et al., 2003a).

As well as demonstrating polarity specific effects, the influence of tDCS has been shown to vary as a function of the duration of stimulation. Transient changes in membrane excitability have been observed during stimulation, where a DC current is administered for short durations in the range of seconds, whereas persistent alterations beyond cessation appear to occur following several minutes of exposure (Stagg and Nitsche, 2011). Neuroimaging and pharmacological interventions have demonstrated that modulations observed at short durations appear to be dependent on changes in the action of sodium and calcium channels (Nitsche et al., 2003b), whereas more persistent adjustments in excitability have been shown to involve the action of *N*-methyl-D-aspartate (NMDA) and γ-aminobutyric acid (GABA) A and B type receptors (Liebetanz et al., 2002; Nitsche et al., 2004a,b, 2005; Tremblay et al., 2013) as well as related changes in the concentration of excitatory and inhibitory neurotransmitters (Stagg et al., 2009, 2011; Clark et al., 2011). As such, the polarity specific effects of tDCS have been compared to long-term potentiation and depression (LTP/LTD) mechanisms (Ridding and Ziemann, 2010; Brunoni et al., 2011; Monte-Silva et al., 2013). However, the majority of studies to date investigating the neurobiological mechanisms underlying tDCS have either not used an explicit behavioral task [instead using motor-evoked potentials (MEPs) to investigate tDCS effects] or have been measured ‘at rest’ (e.g., studies incorporating neuroimaging methods). Those that have used a behavioral task have often employed higher-level cognitive paradigms where it is difficult to conceptualize behavioral change in terms of alterations in membrane potentials.

Of the few studies concerning the effects of tDCS on somatosensory processing, behavioral tactile perception studies have largely focused on Quantitative Sensory Testing (QST; Bachmann et al., 2010; Grundmann et al., 2011; Jürgens et al., 2012) and aspects of spatial discrimination (Ragert et al., 2008; Fujimoto et al., 2014; Yau et al., 2014). tDCS has also been found to modulate vibrotactile frequency discrimination ability, both during and after stimulation (Rogalewski et al., 2004). These studies highlight the links between direct current stimulation and task performance. They do not in general, however, provide a detailed model of the underlying neurobiology supporting the tactile behavior itself. One approach to resolve this problem is to use a behavioral paradigm which is understood more completely at the neurophysiological level and, furthermore, thought to rely upon similar physiological mechanisms to the stimulation method. It would thus be anticipated that integrating tDCS into such an intervention would modify behavioral performance in a predictable manner. By focusing on such paradigms, a more comprehensive understanding of the mechanisms underlying tDCS should result, which could lead to the use of the method in a more optimized way as a potential treatment for neurological and psychiatric disorders (for a review of the clinical applications of tDCS, see Brunoni et al., 2012).

Sensory psychophysics has been used extensively to benchmark links between neurostimulation methods and behavior (Ruff et al., 2006; Lee et al., 2013). In this fashion, we chose to use a vibrotactile adaptation paradigm, where prolonged stimulus exposure has been demonstrated to induce short-term changes in perceptual processing (for an extensive review of vibrotactile adaptation, see Kohn and Whitsel, 2002). The paradigm, known as single-site adaptation (SSA), involves the administration of an adapting stimulus to a single digit (prior to a dual-site amplitude discrimination task) and has been shown to dramatically increase (i.e., worsen) discrimination thresholds (difference limen; DL) compared to those achieved at baseline (Tannan et al., 2007; Zhang et al., 2009). Puts et al. (2013) have recently replicated the expected effect of SSA, demonstrating an average performance decrement of 36% following a 1 s adaptor stimulus. The mechanisms underlying the response to vibrotactile adaptation have been studied extensively in cats and non-human primates (O’Mara et al., 1988; Whitsel et al., 2000, 2003; Chen et al., 2003) and also via electroencephalography in humans (Kelly and Folger, 1999). From this research, it is thought that only the primary somatosensory neurons at the test site are permitted to habituate to the initial adaptor stimulus, which causes a perceptual imbalance in the context of which the two subsequent test stimuli are compared. Due to the reduction in the perceived intensity at the site of the test stimulus, it becomes difficult to distinguish the test from the standard stimulus, which leads to degraded performance compared to baseline. To illustrate this concept, Folger et al. (2008) demonstrated that test stimuli of at least 170 μm would need to be presented to subjects in order to be perceived as different from a standard stimulus of 100 μm, under adaptation conditions. This is in comparison to the baseline condition where, without the influence of adaptation, subjects were capable of discriminating between the 100 μm standard and test stimuli of just 120 μm. Exposure to adaptation stimuli appears to lead to a mismatch between the actual intensity of the stimulus delivered and the subject’s perceptual experience of it, thus preventing finer discriminations due to the mechanisms underlying the reduction in perceived intensity at the test site.

Changes in the concentration of the major inhibitory and excitatory central nervous system (CNS) neurotransmitters, GABA and glutamate, as well as the action of related post-synaptic receptors (GABA_A_, NMDA) have been suggested to underlie vibrotactile adaptation (Lee and Whitsel, 1992; Lee et al., 1992). The role of the excitation/inhibition balance has also been emphasized during subsequent animal-based, optical intrinsic signal (OIS) imaging investigations, in which local competitive interactions between minicolumns appear to be essential in molding the response to repetitive stimuli (Tommerdahl et al., 2002; Chiu et al., 2005; Simons et al., 2005, 2007). Accordingly, increased absorbance (a marker of neuronal activity) at the site of the stimulus has been observed alongside inhibition of the surrounding region, supporting the role of GABAergic, lateral inhibition in vibrotactile adaptation. The proposed role of GABAergic inhibition has also been determined via the assessment of performance in a range of human subject populations, including those with Autism Spectrum Disorder (ASD), concussion, migraines, and alcohol dependence (Tannan et al., 2008; Zhang et al., 2011; Nguyen et al., 2013a,b). The expected SSA effect demonstrated by healthy controls is notably absent in these samples, despite achieving largely similar baseline scores. This discrepancy in performance is thought to emerge from the presence of altered CNS sensitivity in the respective samples. For example, evidence suggests that individuals with ASD are likely to exhibit abnormal cortical excitability levels due to a reduction in inhibitory neurotransmission (Casanova et al., 2002, 2003; Uhlhaas and Singer, 2006, 2012). This suggests that a loss of normal inhibitory function – possibly mediated by GABAergic mechanisms – is likely to produce an atypical response to adaptation. The work of Folger et al. (2008) extends support for the interpretation of the clinical population studies. Healthy control subjects given Dextromethorphan (DXM), an NMDA receptor (NMDAR) antagonist, were found to achieve similar baseline performance to those given a placebo but failed to demonstrate the usual decline in SSA performance. The action of DXM was suggested to facilitate a release from inhibition: NMDAR activation has been shown to provide a significant drive in facilitating GABAergic transmission in interneurons (Xue et al., 2011), meaning its blockade should greatly reduce inhibitory transmission. Importantly, such a reduction in GABAergic ‘tone’ has been proposed to underlie the effects of anodal tDCS (Stagg et al., 2009; in addition to the established role of glutamatergic, NMDARs; Nitsche et al., 2003b). On the basis of the outlined GABA modulation, comparable results to the DXM study may be obtained post-stimulation. Therefore, the neurobiological similarity between vibrotactile adaptation and tDCS meant that the paradigm represented an ideal starting point from which to examine the proposed GABAergic contribution to the effects of tDCS.

Integrating anodal and sham tDCS into the vibrotactile amplitude discrimination paradigm, predictions were that active tDCS would not produce changes in discrimination thresholds for the baseline task whereas a decrease in threshold values (i.e., an improvement) would be observed for the SSA condition. In the presence of anodal stimulation, it was proposed that resting membrane potential would be elevated (via a release from inhibition; increased NMDA efficiency and decreased GABAergic neurotransmission) such that the cortical excitability profile of subjects should mimic that proposed for individuals with altered CNS sensitivity. While baseline thresholds in such populations have been established as similar to those of healthy controls, such individuals do not appear to be susceptible to the influence of adapting stimuli. Therefore, during the adaptation version of the task, subjects were predicted to obtain better performance measures (lower discrimination thresholds) following anodal compared to sham tDCS.

## Materials and Methods

### Subjects

To determine the ideal sample size, baseline and adaptation values from several studies were acquired from the lab of a collaborator (Prof. Mark Tommerdahl). Power calculations were performed using G^∗^Power Version 3.1 (University of Dusseldorf, Germany; Faul et al., 2007). Sample sizes ranging from *N* = 9 to *N* = 23 have been quoted in the SSA literature; however, based on the effect size of the existing data the calculations recommended a sample size of *N* = 9 (assuming one-tailed significance). It is important to note that the sample size was foremost calculated to ensure a behavioral effect as opposed to a behavior^∗^tDCS interaction. Additionally, use of the behavioral data was necessary in the absence of adequate tDCS data on which to base sample size calculations, due to the novelty of the study. However, many studies incorporating tDCS have used such sample sizes and achieved significant modulations of their selected paradigms (Elmer et al., 2009; Ladeira et al., 2011; Spiegel et al., 2012; Tang and Hammond, 2013; Pavlova et al., 2014).

Twelve participants took part in the study (seven female). Subjects were aged 19–31 years (*M* = 24.08, *SD* = 3.34) and right-hand dominant, determined by the short-form Edinburgh Handedness Inventory (Oldfield, 1971). Upon expressing an interest in taking part in the study, subjects were issued with a screening form to determine their eligibility. Those with any contraindications were excluded from the study. Subjects gave their written, informed consent prior to participating and all procedures were carried out with the approval of the local ethics committee.

### Vibrotactile Task

Subjects completed two versions of a 2AFC task, designed to test their ability to discriminate between vibratory stimuli of differing amplitudes. Stimuli were delivered to the index and middle finger (digits 2 and 3) of the left hand, using a vibrotactile stimulation device capable of delivering dual-site stimuli (CM5; Cortical Metrics, Chapel Hill, NC, USA). The probe tips on the device were 5 mm in diameter.

**FIGURE 1:**
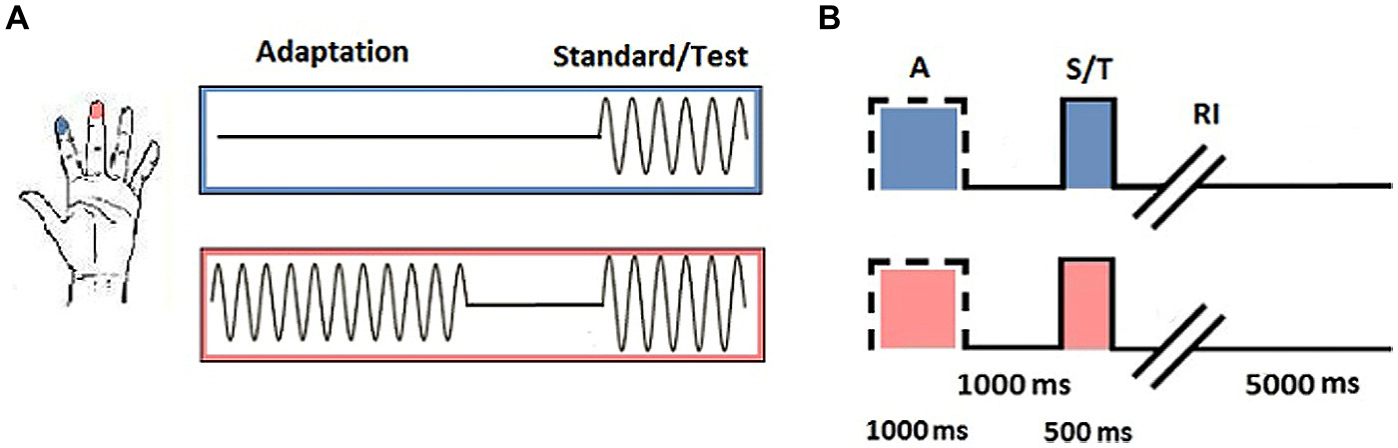
**Vibrotactile trials. (A)** Trial stimulation: 25 Hz sinusoidal stimuli were delivered to D2 and D3 of the left hand. Adaptation trials consisted of a single pulse delivered to one digit (in this instance D3). During the test phase, stimuli were delivered simultaneously to D2 and D3. Subjects were required to determine which stimulus was of the highest amplitude. Baseline trials consisted only of the test phase. **(B)** Trial timing: Adaptation trials began with the presentation of a single pulse to the selected digit (A; 1000 ms), followed by an interval between the adaptor and test stimuli (1000 ms) before the standard and test stimuli were simultaneously delivered (S/T; 500 ms). Subjects were given an unrestricted response interval (RI) to indicate which digit they thought had received the stimulus of highest amplitude, after which an interval signaled the onset of the next trial (5000 ms; figure adapted from Tannan et al., 2007, with permission).

Each subject completed baseline and SSA runs. During the baseline task, subjects were asked to determine which of two simultaneously delivered stimuli felt more intense. In the SSA task, subjects were instructed to ignore a single vibration before making the same intensity judgment on the subsequent pair (see **Figure 1A** for a schematic representation of each phase of the task).

Responses on each task were tracked using a staircase method (reviewed in Leek, 2001). The first half of trials was executed in a 1up/1down protocol, whereby one correct or incorrect response was sufficient to signal a decline or enhancement in performance. The amplitude of the test stimulus selected for the subsequent trial was adjusted in accordance with the response accuracy of the previous trial. The final half of trials was conducted using a 2up/1down protocol, in which two correct responses were required before performance was classified to have improved and the amplitude of the test stimulus was reduced. Step size was maintained at 20 μm across all trials and experimental runs. All vibrotactile pulses were sinusoidal and were delivered at 25 Hz (defined as flutter stimulation). Adaptor amplitude was 200 μm, which was identical to that of the proceeding standard stimulus. The test stimulus varied between 205–400 μm. The duration of the adaptor was 1000 ms, with a 500 ms interval for the test phase. Standard and test pulses were delivered simultaneously. The location of the standard and test stimuli was randomized across trials.

### Transcranial Direct Current Stimulation

**FIGURE 2:**
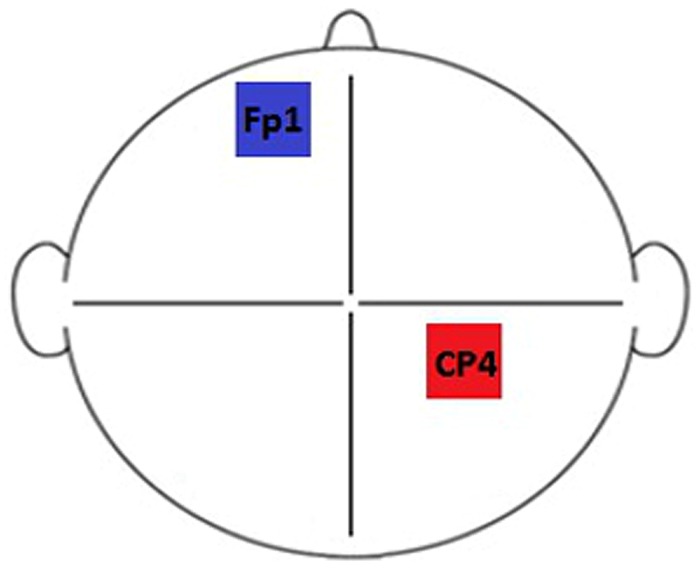
**Electrode montage.** Electrodes were positioned at locations CP4 (right hemisphere/contralateral to the stimulus, anode) and Fp1 (left hemisphere, cathode) of the 10–10 system.

Brain stimulation was delivered via a DC-Stimulator Plus device (neuroConn, Germany). Subjects participated in two sessions defined by stimulation type: Anodal (A) and Sham (S). Each session took place (at least) 1 week apart. The experiment was double-blind: both the researcher and the subjects were naive to the nature of the stimulation that took place during each session. Stimulation duration was set to 600 s for each session, with a 10 s current fade in/out period. Rubber electrodes, measuring 5 cm × 7 cm (35 cm × 35 cm), enclosed in saline soaked sponges (0.9% concentration) were used to deliver anodal stimulation with a current of 1 mA (current density = 0.029 mA/cm^2^). For sham stimulation, the current was ramped up to mimic the peripheral effects of tDCS before being ramped down automatically. During the course of the designated stimulation period, the device continued to discharge minute current spikes every 550 ms (110 μA over 15 ms) to enable continuous impedance readings. The average current over time was not more than 2 μA, which the neuroConn device manual describes as having no therapeutic effect. A somatosensory/contralateral orbit montage was selected as the most commonly used configuration for somatosensory stimulation paradigms (based on the studies listed in Nitsche et al., 2008). Electrodes were positioned using the 10–10 system at landmarks Fp1 (left hemisphere, cathode) and CP4 (right hemisphere/contralateral to the stimulus, anode), designed to correspond to primary somatosensory cortex (Chatrian et al., 1985). The electrode configuration used is illustrated in **Figure 2**.

### Experimental Procedure

Subjects were seated in front of a computer monitor with the vibrotactile stimulation device positioned on their left-hand side. They were instructed to lightly rest their digit tips over the corresponding finger pads. Adaptation trials began with an initial period of single-site stimulation, which was to be ignored by subjects. This was followed by an interval before the test phase, in which a period of dual-site stimulation was delivered. After the test stimuli had been presented, subjects had an unrestricted amount of time to make the required intensity discrimination. Baseline trials incorporated only the test phase (see **Figure 1B** for a schematic representation of stimulus timing). Subjects responded with their right-hand, using the left and right mouse buttons. A left click corresponded to D3 and a right click corresponded to D2. Subjects were provided with visual cues to guide their responses. These were in the form of “IGNORE!” and “TEST!” statements that appeared on screen during the respective stimulation periods.

**FIGURE 3:**
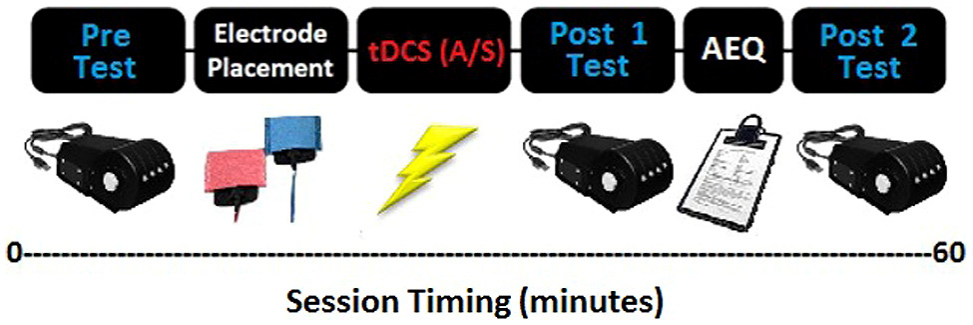
**Experimental design.** Subjects initially completed one run of each of the vibrotactile tasks before receiving anodal or sham stimulation. This was followed by another two blocks of the tasks, post-stimulation. Subjects completed an adverse effects questionnaire between the post-stimulation task blocks.

Subjects began each session by completing one block of the vibrotactile tasks (20 trials per block). After completing the initial runs, subjects were prepared for tDCS (administered as previously outlined). The presentation of each task and stimulation type was fully counterbalanced. Following DC stimulation, two more blocks of the vibrotactile tasks were completed. The first block took place 5 min after stimulation had terminated (5–15 min post-tDCS; Post 1) and the second block was executed after 20 min had elapsed since the end of stimulation (20–30 min post-tDCS; Post 2). The first post-tDCS block was designed to detect the presence of tDCS after-effects while the second block was included to gain insight into the persistence of any such evident effects. Between the first and second post-tDCS blocks, subjects answered an adverse effects questionnaire (AEQ) to assess the presence of any side-effects related to stimulation (see *Supplementary Material* for the AEQ items). Subjects were also given the questionnaire before each subsequent session to assess side-effects of prolonged duration and/or delayed onset. Experimental sessions lasted approximately 60 min in total (see **Figure 3** for a chronological overview of session timing).

### Data Analysis

The data were plotted using MATLAB (Version 7.4.0; MathWorks, Cambridge) to derive performance curves for each experimental run. These were visually inspected for evidence of threshold stabilization and adequate performance capability (standard task progression and final DL values). Excessive noise in the data constituted grounds for exclusion. The majority of subjects’ performance curves were satisfactory, however, data from two subjects was declared unsuitable for future analysis. These subjects were subsequently removed and two additional subjects, of a similar demographic to the initial subjects, were recruited to keep the design counterbalanced: 12 subjects (seven female), aged 19–31 years (*M* = 23.50, SD = 3.63). Statistical analyses were computed using SPSS for Windows software (Version 20; IBM, New York, NY, USA). Data were initially compared with regard to differences between pre-tDCS, baseline and adaptation trials. The DL value for each run, representing the average test stimulus value from the final five trials, was entered separately into a two-way, Repeated Measures ANOVA analysis with the following variables: Condition (Baseline, SSA) and Session (1, 2). Subsequently, to assess the influence of tDCS stimulation across time and conditions, scores were entered into a three-way, Repeated Measures ANOVA, including the following variables: Condition (Baseline, SSA), tDCS (Anodal, Sham) and Time (Pre, Post 1, Post 2). The between-subject factor tDCS order (Anodal/Sham, Sham/Anodal) was also incorporated into the post-tDCS analysis. Where appropriate, Greenhouse–Geisser correction was used to compensate for violations of sphericity. An alpha level of 0.05 was used to determine significance.

## Results

### Pre-tDCS Data

**FIGURE 4:**
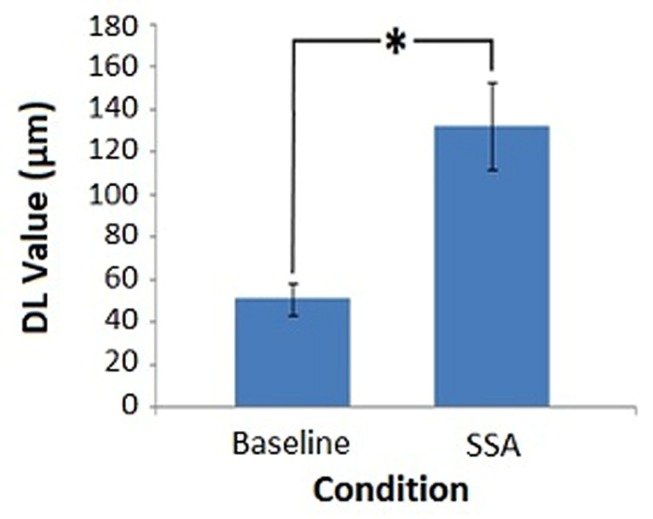
**Pre-tDCS amplitude discrimination thresholds.** Average DL values for each task condition, obtained prior to DC stimulation (^∗^denotes significance, *p* < 0.05). Error bars represent ±1 SEM.

Average DL values were computed across subjects for each condition: Baseline Session 1 (*M* = 56.58, *SD* = 30.30), Baseline Session 2 (*M* = 45.25, *SD* = 24.20), SSA Session 1 (*M* = 139.67, *SD* = 78.98), SSA Session 2 (*M* = 125.00, *SD* = 64.59). The overall pre-tDCS mean values were also calculated by averaging data across sessions (**Figure 4**), in order to demonstrate the distinction in DL values between conditions. The lowest amplitude discrimination thresholds appear to have been achieved during the baseline condition. In comparison, threshold values obtained during the SSA condition were not only higher but were also more variable. Furthermore, the mean values for each run indicate that DL values resulting from the first session, for each condition, were higher than those of the second session. For this reason, the data was entered into a Repeated Measures ANOVA to assess the potential influence of repeated task exposure.

The 2 × 2 ANOVA produced a significant main effect for Condition [*F*(1,11) = 35.484, *MSE* = 2241.674, *p* = 0.000]. The main effect of Session failed to reach significance [*F*(1,11) = 0.718, *MSE* = 2825.227, *p* = 0.415]. As did the interaction between Condition and Session [*F*(1,11) = 0.018, *MSE* = 1861.833, *p* = 0.896]. The results in relation to Condition were as expected: the SSA condition threshold values were significantly higher than those of the baseline measure. The lack of significant difference between sessions indicated that repeat exposure to the tasks did not produce a substantial change in the threshold values obtained.

### Post-tDCS Data

During stimulation, impedance levels were on average 6.27 kΩ: Anodal (*M* = 6.61, *SD* = 2.84), Sham (*M* = 5.93, *SD* = 1.71). Subjects reported minor adverse effects, including mild to moderate itching and tingling sensations underneath the electrodes. Slight tiredness and difficulty concentrating were also documented, as was a mild burning sensation at current onset. In the period following stimulation, mild itching, tingling, tiredness, and difficulty concentrating were reported. While peripheral sensations are commonly observed, the concentration problems documented suggest that the reference electrode may have affected the excitability of frontal regions (Nitsche et al., 2005). A single subject also described the atypical incidence of a warming sensation to their upper body and a change in mood described as a general feeling of contentment and relaxation. Only mild itching and tiredness persisted beyond the end of each session and all subjects responded positively to participating in further tDCS studies (see *Supplementary Material* for a summary of AEQ responses). A series of Paired Samples *t*-tests determined that subjects experienced similar sensations during anodal and sham stimulation for the side effects reported; Tingling [*t*(11) = 0.616, *p* = 0.551], Itching [*t*(11) = 2.171, *p* = 0.053], Burning [*t*(11) = 0.561, *p* = 0.586], Pain [*t*(11) = 1.000, *p* = 0.339]; Vision problems [*t*(11) = –1.000, *p* = 0.339], Concentration problems [*t*(11) = 1.393, *p* = 0.191], Tiredness [*t*(11) = 1.000, *p* = 0.339]. Therefore, subjects were likely not aware of whether they received sham or active stimulation based solely on peripheral sensations.

**FIGURE 5:**
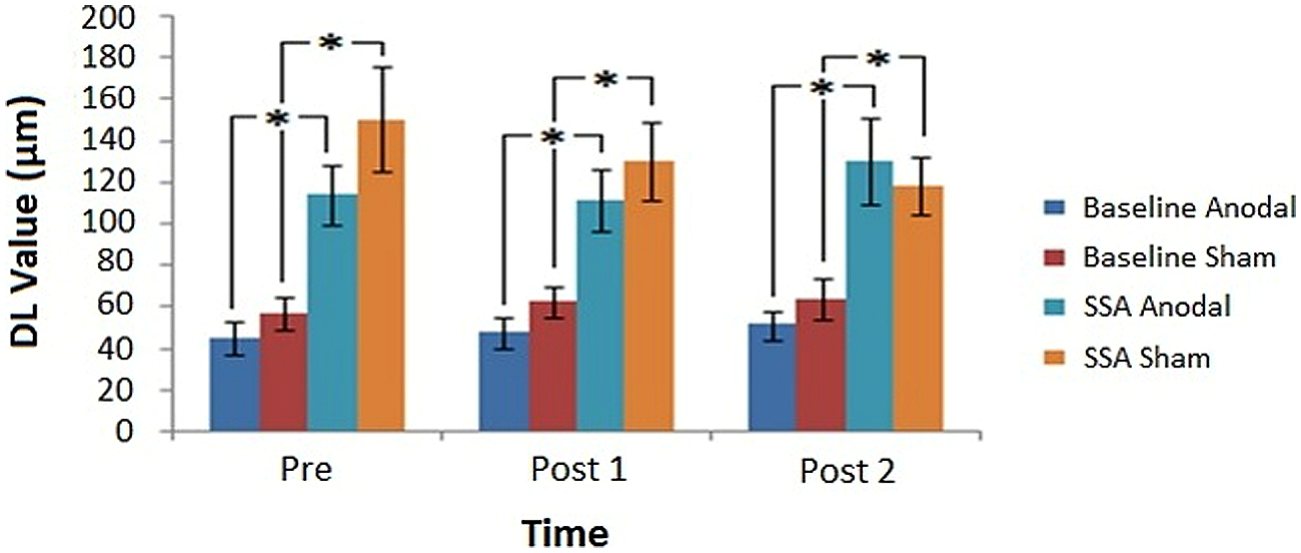
**Post-tDCS amplitude discrimination thresholds.** Average DL values obtained before and after tDCS, for each task condition in relation to the assessed stimulation modes (^∗^denotes significance, *p* < 0.05). Error bars represent ±1 SEM.

Average DL values were computed across subjects for each condition, across tDCS and Time (**Figure 5**). **Figure 5** illustrates that the SSA thresholds were consistently higher than those of the baseline measure. In relation to DC stimulation, general trends in the data indicated that anodal and sham baseline measures were stable, anodal SSA values were similar for Pre and Post 1 runs but increased at the Post 2 time point, whereas sham SSA values declined slightly over time.

The 2 × 2 × 3 Repeated Measures ANOVA established a significant main effect for Condition [*F*(1,10) = 56.223, *MSE* = 3263.261, *p* = 0.00002]. The main effects of tDCS [*F*(1,10) = 2.227, *MSE* = 3031.556, *p* = 0.166] and Time [*F*(2,20) = 0.165, *MSE* = 1125.478, *p* = 0.849] failed to reach significance, as did the associated within-subject variable interactions. The influence of the between-subjects factor Gender did not meet the criteria for significance [*F*(1,10) = 1.337, *MSE* = 8869.840, *p* = 0.275]. However, tDCS order was found to be significant [*F*(1,10) = 9.253, *MSE* = 5222.744, *p* = 0.012], as was the Condition^∗^tDCS order interaction [*F*(1,10) = 8.175, *p* = 0.017]. The interaction appeared to stem from a general tendency for DL values to be lower when subjects experienced anodal prior to sham stimulation, which was particularly evident for the SSA scores. This tendency was further tested using a Repeated Measures ANOVA with factors of Time (Pre, Post 1, and Post 2), tDCS order (Anodal/Sham, Sham/Anodal) and Session (1, 2) to analyze the task conditions separately. A significant main effect of tDCS order was demonstrated only for the SSA condition [Baseline (*F*(1,5) = 2.216, *MSE* = 720.425, *p* = 0.197); SSA (*F*(1,5) = 7.590, *MSE* = 9671.414, *p* = 0.040)]. These results confirmed the previous suggestion that only the data from the SSA condition was significantly influenced by stimulation order. The main effect of Session for each of the conditions was non-significant [Baseline (*F*(1,5) = 3.883, *MSE* = 476.514, *p* = 0.106); SSA (*F*(1,5) = 0.251, *MSE* = 4241.014, *p* = 0.637)]. Additionally, analysis of the SSA condition resulted in a Time^∗^tDCS order^∗^Session interaction that narrowly failed to reach significance [*F*(2,10) = 4.049, *MSE* = 898.747, *p* = 0.051]. While DL values did not appear to have fluctuated between sessions in general (for either of the conditions), the interaction found for the SSA data suggests that there may have been a more subtle influence of tDCS order at a specific time point for a particular session. Such an outcome may manifest as a carry-over effect, in which the influence of tDCS order (particularly A/S) would be shown to lead to a distinction in DL scores across sessions.

Although the non-significant main effect of Session for both conditions suggested the influence of repeated task exposure could be ruled out, any such carry-over effect could still be largely confounded by familiarity with the task. To address the trend established by the interaction, while minimizing the effect of practice, scores from the Pre time point were assessed across sessions for each tDCS order. This approach allowed for insight into task performance prior to any stimulation in the first session, while assessing any residual effect of having previously undergone a single application of either Anodal (A/S group) or Sham (S/A group) tDCS at the start of the second session. Paired Samples *t*-tests revealed a significant difference in subjects’ DL values for the SSA condition during the first session, which corresponded to lower scores for the A/S order [*t*(5) = –2.695, *p* = 0.043]. This indicated a pre-existing difference in performance, irrespective of tDCS stimulation, most likely illustrating initial ability to execute the task. The same comparison performed on data from session 2, following a single application of tDCS, was found to be non-significant [*t*(5) = –0.671, *p* = 0.532]. Therefore, each group produced statistically similar thresholds at the Pre time point during session 2. Assessing each stimulation order separately, neither groups’ performance altered between sessions: A/S [*t*(5) = –0.524, *p* = 0.622]; S/A [*t*(5) = 1.975, *p* = 0.105]. This illustrates that the stimulation given in the first session was unlikely to have influenced scores during the second session, thus opposing the existence of a carry-over effect.

To summarize, the results indicate that significantly higher thresholds were consistently produced during the SSA condition compared to the baseline task, which parallels the findings present in the pre-tDCS analysis. The non-significant main effect of tDCS demonstrates that subjects’ DL values did not significantly change between anodal and sham sessions. However, the significance of the between-subjects variable tDCS order and the Condition^∗^tDCS order interaction suggests that lower thresholds resulted during the SSA condition when subjects were exposed to anodal prior to sham stimulation (albeit in the absence of a carry-over effect).

While traditional, frequentist statistics permit the acceptance of experimental hypotheses, where criteria for a significant *p*-value have been fulfilled, they do not allow for valid inferences to be made on the acceptance of the null hypothesis (in light of established non-significant differences between conditions: Wagenmakers, 2007; Kruschke, 2010; Dienes, 2011). Such support for the null hypothesis can be derived using Bayesian statistics (Dienes, 2014), an approach which has become increasingly popular in recent years (for examples of use, see Verbruggen et al., 2013; Greve et al., 2014). Opposing models, typically representing the experimental and null hypotheses, are compared to generate a Bayes factor (*B*), which constitutes a ratio of the likelihood of each model being true. By computing a Bayes factor, one of three outcomes can be achieved based on the generated value. It is common to interpret these as follows: a *B*-value of less than a third corresponds to strong support for the null hypothesis; a value of between a third and 3 relates to insubstantial evidence for a firm conclusion; and values above 3 indicate evidence for the alternative hypothesis (Jeffreys, 1961). Therefore, a Bayesian analysis framework was adopted to investigate whether the results of the current study genuinely reflected that tDCS had no effect on task performance. This was specifically targeted toward the SSA condition, where the alternative hypothesis stated that a decrease in discrimination thresholds should have been evident following anodal compared to sham stimulation.

A half-normal distribution model was chosen in light of the directionality of the prediction (Dienes, 2014). The model specifies that the theoretical variance for the population can be estimated (e.g., establishing a value to represent the standard deviation of a given sample). While the effect size of tDCS has previously been shown to be similar in magnitude to that of a corresponding behavioral intervention (Harty et al., 2014), it does not seem reasonable to expect that the application of tDCS should be as effective as the difference between behavioral conditions in all instances (considering tDCS-driven effect sizes appear to be mediated by factors such as electrode placement and montage selection: Mathys et al., 2010; Schambra et al., 2011). In the absence of existing tDCS effect size data for the vibrotactile paradigm, the present study estimated that a tDCS modulation of the behavioral effect would be equivalent to half the magnitude of the established mean behavioral difference (between the SSA and baseline task conditions). To reduce the results into a single vector, the Post 2 data was removed to facilitate a more simplistic pre/post design (having proposed that any observed tDCS influence would be most evident as a distinction between the Pre and Post 1 as opposed to Pre and Post 2 runs).

The data was assessed using the MATLAB version of an online Bayes calculator (http://www.lifesci.sussex.ac.uk/home/Zoltan_Dienes/inference/bayes_factor.swf). Sample mean (*M* = –17.75) and sample size corrected, standard error values (*SEM* = 21.40) were calculated. The population mean was set to zero and the likely population standard deviation was defined as being half that of the observed behavioral effect size (as specified above). This was derived from the pre-tDCS data as the mean difference of the grand average SSA value and that of the baseline condition [(132.33-50.92)/2 = 40.71]. The corresponding Bayes factor was 0.28. The analysis indicated strong support for the null hypothesis that tDCS did not have an effect on the performance of the vibrotactile adaptation task.

## Discussion

The current research aimed to investigate the role of modifications in cortical plasticity on amplitude discrimination performance, with the wider aim of further investigating the physiological underpinnings of tDCS after-effects. As shown in the existing literature, the pre-tDCS results indicated that in the presence of adaptation stimuli, amplitude discrimination thresholds were vastly degraded compared to baseline trials. However, no changes in threshold were established following the application of anodal tDCS.

### Pre-tDCS Findings

The results of the pre-tDCS analysis provide supporting evidence that the presence of a short duration, adaptation stimulus was sufficient to produce changes in behavioral performance in our vibrotactile task. SSA scores were significantly higher than those at baseline. This finding parallels those of other studies using healthy control subjects to investigate the influence of SSA on amplitude discrimination (Tannan et al., 2007; Zhang et al., 2009; Puts et al., 2013). Compared to the results of Puts et al. (2013), in which a 36% difference between baseline and SSA thresholds was established, a percentage difference of 62% was derived from the current study. Puts et al. (2013) adopted an adaptor amplitude of 100 μm compared to the 200 μm adaptor stimulus incorporated into the current study, which may explain the variation in difference measures in terms of what is known about the influence of stimulus intensity. The magnitude of the adaptation response has been shown to vary as a product of adaptor amplitude. From a physiological perspective, stimuli of heightened amplitude produce more pronounced cortical responses (Chiu et al., 2005). During an optical imaging study, Simons et al. (2005) discovered increased absorbance (increased firing rate) at regions 3b and 1 of primary somatosensory cortex following 400 μm compared to 50 μm stimulation. Although the spatial extent of activation remained the same, a decrease in absorbance was detected at neighboring regions. This corresponded to the lateral inhibition of unrelated neuronal populations via an increase in the responsiveness of GABAergic processes. By using a higher amplitude adaptor stimulus in the present study, there was a potential for the resulting reduction in perceived intensity to be more dramatic than previously established. Therefore, an increase in the prominence of the observed adaptation effect was to be anticipated due to a resulting increase in the magnitude of cortical response elicited, as well as the better defined development of an inhibitory surround area.

### Post-tDCS Findings

The SSA studies conducted using clinical populations (e.g., ASD; Tannan et al., 2008) and pharmacological interventions (e.g., DXM; Folger et al., 2008) emphasize the role of inhibitory processing in adaptation performance. It is common for ASD to be investigated from the perspective of CNS hyperexcitability, which may be driven by abnormal minicolumn structure and impaired GABAergic inhibition (Casanova et al., 2002, 2003; Uhlhaas and Singer, 2006, 2012). Similarly, while the administration of DXM provides insight into hypoexcitability via reduced NMDAR efficiency, the resulting reduction of excitation also leads to a decrease in the recruitment of associated inhibitory processes (Xue et al., 2011). Therefore, both sets of studies infer that reduced inhibition is integral to the finding that those with altered CNS sensitivity do not respond to the presence of adapting stimuli in the typical manner. As anodal tDCS is also thought to be dependent on an alteration of the efficacy of inhibitory, GABAergic mechanisms (Stagg and Nitsche, 2011), performance changes following tDCS were expected to occur in line with those who are GABA deficient. While no modulation of discrimination thresholds was observed for the baseline task (as predicted), anodal tDCS was also demonstrated to have no influence on SSA performance, compared to sham.

Although the significant influence of tDCS order suggests that lower thresholds were established when subjects’ were exposed to anodal stimulation in their first session, this is unlikely to be a product of the stimulation itself. Instead, it may be more plausibly explained by the variation in thresholds for the SSA condition. For example, with regard to the potential carry-over effect, the S/A group scores decreased between sessions (DL 188.67–137.33), which would not be expected for the sham group because it represents an inactive mode of stimulation. However, the opposite pattern was observed for the A/S group, where scores increased (DL 90.67–112.67). General practice effects that could be used to interpret the S/A group decrease did not emerge in the A/S group as might be expected, meaning the results are most likely due to the instability of SSA scores. The implementation of Bayesian statistics allowed for further insight into the non-significant effect of DC stimulation by resulting in a Bayes factor that provided substantial evidence for the null hypothesis. This implies that there were no differences between thresholds derived following anodal compared to sham stimulation for the SSA condition. Additionally, despite the sample size being optimized toward the detection of the desired behavioral effect, the outcome of the Bayesian analysis suggests that the study was sufficiently powered to provide substantial evidence with regard to the outcome of the tDCS intervention.

The question remains that, considering the substantial overlap in the proposed physiological mechanisms of anodal tDCS and vibrotactile adaptation – why were there no changes in the observed thresholds, to the extent that the null hypothesis could be supported? Crucially, do the proposed mechanisms underlying tDCS, or perhaps those relating to the vibrotactile task, need to be revised? Focusing on the efficacy of the stimulation method itself, there are several factors which may have contributed to the lack of observed tDCS effect on amplitude discrimination performance. Individual differences have been shown to influence cortical plasticity, which may create possible sources of variance and dramatically impact upon results (Ridding and Ziemann, 2010). In a recent study, Wiethoff et al. (2014) determined that approximately 75% of responses to anodal tDCS, delivered to motor cortex, were facilitatory but the remaining responses were of an inhibitory nature. While variability will inevitably differ between studies for many reasons (e.g., those related to the stimulation protocol), such inter-subject variation may present a significant confound such that analysis on an individual rather than group level may be warranted (as illustrated in a recent review; Horvath et al., 2014). Several studies investigating the influence of tDCS on responses to QST further emphasize the impact of inter-subject variation (Bachmann et al., 2010; Grundmann et al., 2011; Jürgens et al., 2012). Despite incorporating similar sample populations and sensorimotor montages as well as identical current intensities, durations, and electrode sizes, the results of each study differed dramatically. A potential source of variance is represented by the unique nature of an individual’s brain anatomy, which is likely to produce differences in the current density levels at the target brain region (Russell et al., 2013; Kim et al., 2014a). A recent simulation study has illustrated that the highest current densities are likely produced 2–4 cm from the target region under the electrodes, falling in a region between the active and reference sites (Rampersad et al., 2014). In relation to the montage adopted as part of the current study, peak current strength may have been situated over the Vertex (Cz) which could explain the lack of tDCS effects as this region is presumed to be functionally inert. Although establishing realistic head models of current pathways is computationally demanding, these studies highlight their importance when considering the influence of tDCS on task performance.

The results may also have been confounded due to gender differences. Research suggests that females are susceptible to hormone fluctuations linked to GABAergic neurotransmission levels and that there is a general stability of crucial excitatory and inhibitory processes in men compared to women (Kuo et al., 2006; Chaieb et al., 2008). At specific points of the menstrual cycle, females experience stages of greater GABAergic neurotransmission via increased progesterone levels (Epperson et al., 2002; Smith et al., 2002). This fluctuation in GABA levels was not controlled for as part of the study. Recruiting females at different cycle stages may have led to any general neuromodulatory effects being canceled out. This would be further compounded when coupled with the GABA stability in male subjects, who may elicit an attenuated response compared to females. Gender effects were assessed as part of the current research and were not found to produce significant differences in performance, although the small size of the groups compared makes it difficult to derive substantial inferences. Future research to define the nature of tDCS after-effects in both male and female-only samples is needed to clarify whether sex differences present a realistic confound.

The impact of genetic contributions on the efficacy of neurostimulation techniques has previously been demonstrated. Brain-Derived Neurotrophic Factor (BDNF) has been implicated in LTP and has a profound effect on pre-synaptic glutamate release and post-synaptic NMDAR function (Nathan et al., 2011). Accordingly, the presence of the Val66Met polymorphism, which impairs the action of BDNF and therefore glutamate release, has been proposed to influence cortical plasticity. This could dramatically impact on the effect of stimulation methods, such as rTMS and tDCS (Cheeran et al., 2008; Fritsch et al., 2010), although not all studies have reported the expected detrimental association between the Val66Met polymorphism and tDCS-induced plasticity (Antal et al., 2010). Additional factors that have been reported to affect cortical plasticity include advancing age, which reduces plasticity (Fathi et al., 2010) and regular exercise, which increases plasticity (Cirillo et al., 2009). The time of day at which subjects are tested may also produce variable effects with evidence suggesting that cortical plasticity is enhanced in the afternoon (Sale et al., 2007). However, a Magnetic Resonance Spectroscopy (MRS) study on the influence of time of day on GABA levels failed to document any significant fluctuations (Evans et al., 2010), indicating that altered cortical plasticity may not primarily be due to the measure of GABAergic inhibition derived by MRS (e.g., related specifically to neurotransmission; Stagg, 2014).

It is also entirely plausible that while the influence of tDCS was not visible at a behavioral level, physiological changes may have still been induced. Suntrup et al. (2013) demonstrated a significant event-related desynchronization (ERD) in the theta band, following tDCS, for a task of moderate difficulty. However, the study failed to establish any pre/post differences using a behavioral metric for the same task. Only the more difficult version of the task produced significant behavioral results. As the current study did not measure any index of physiological modulation it is impossible to draw conclusions as to whether such alterations took place. Nonetheless, it appears that the nature of the task as well as its inherent level of difficulty may not have been sufficient for tDCS modulation to occur at the level of behavior. In relation to task difficulty, the use of a staircase method prevents researcher control of task difficulty because stimulus presentation is based entirely on an individual’s performance, which is unique to their particular threshold. Although establishing an individual’s threshold should involve trials becoming progressively more difficult, those who perform well will inevitably experience the tasks as less challenging thus creating a bias in the sample. Use of a method of constant stimuli approach (Leek, 2001), in which the stimuli to be presented are set *a priori*, may offer some insight into the role of task difficulty.

With regard to the nature of the task used in the current study, it may be possible that the use of a purely perceptual task without an explicit learning component may have contributed to the lack of an observed tDCS effect. The use of anodal stimulation protocols coupled with motor learning tasks has highlighted the potential of the neuromodulation technique to induce predictable, performance enhancements (Nitsche et al., 2003a; Stagg et al., 2011; Kim et al., 2014b). While such tasks have been hypothesized to recruit LTP-like mechanisms, research into the underpinnings of amplitude discrimination has primarily focused on altered lateral inhibition processes (Tommerdahl et al., 2002; Chiu et al., 2005). However, a balance between glutamatergic excitation and GABAergic inhibition is required for LTP to take place (Trepel and Racine, 2000). Although the close biochemical coupling of GABA and glutamate suggests that a change in GABA is likely to be accompanied by a correlated change in glutamate, the precise role of glutamatergic neurotransmission in amplitude discrimination is largely unknown. Like the aforementioned motor learning tasks, *in vitro* responses to repetitive stimulation have also been characterized in terms of LTP (Lee et al., 1992). Supporting findings have been established *in vivo*, where NMDAR antagonists were able to attenuate the cortical adaptation response (Lee and Whitsel, 1992). However, the stimuli used in relation to these animal studies were directly delivered to the brain and were in the duration of minutes rather than seconds as commonly used in modern human studies. It may, therefore, be the case that the necessary reduction in inhibition and parallel increase in NMDAR efficiency may not have occurred during the task as performed in human subjects, making it difficult for tDCS to modify behavioral performance.

Finally, it may have been the case that aspects of the adopted stimulation protocol may have prevented the emergence of a tDCS effect. Rogalewski et al. (2004) demonstrated a modulation of performance on a vibrotactile frequency discrimination task via cathodal stimulation, while no effect of anodal stimulation was observed. The present study did not employ cathodal stimulation and as such may have failed to report a stimulation-induced alteration in task performance for this reason. Additionally, Rogalewski et al. (2004) demonstrated that the observed reduction in correct responses related to cathodal tDCS began during the stimulation period and Ragert et al. (2008) established a facilitatory effect of anodal tDCS on spatial discrimination ability, which also emerged during the stimulation period. There is much debate with regard to the ideal point at which to administer tDCS. Quartarone et al. (2004) demonstrated that the completion of a motor imagery task, after DC stimulation, was able to diminish the influence of anodal tDCS but extended that of cathodal tDCS. Conversely, Antal et al. (2007) reported a reversal of the expected influence of anodal stimulation, thus mimicking cathodal effects, following the performance of a simple motor task administered during stimulation. Therefore, to boost the likelihood of inducing plasticity changes, it has yet to be established whether it is advantageous to attempt to synchronize the onset of stimulation with that of the task by adopting an “online” stimulation approach. An alternative may be to alter the typical pre/post stimulation pattern. For example, Bastani and Jaberzadeh (2014) reported that several within-session doses of tDCS, with extended intervals between each subsequent exposure, may be the key to enhancing cortical excitability. Nonetheless, as this study employed a classic pre/post design, such potential effects may have been missed. Lastly, it is of relevance that the expected duration of effects following somatosensory stimulation has also yet to be determined. This makes it difficult to know what constitutes a typical response, such that establishing the optimal structure of post-stimulation task blocks is problematic. For example, Rogalewski et al. (2004) demonstrated short-lasting after effects of 7 min, whereas those observed by Ragert et al. (2008) persisted for 40 min. The distinction between these studies may reflect differences in stimulation duration, polarity, and/or current density; the effects of which are less appreciated outside of motor cortex (as discussed by Chaieb et al., 2008).

## Conclusion

The study supported previous findings that SSA stimuli are capable of producing robust decrements in vibrotactile amplitude discrimination thresholds, compared to those established at baseline. Despite evidence suggesting a similarity in terms of the neurobiological mechanisms underlying our behavioral and neurostimulation methods, anodal stimulation was unable to modulate the observed adaptation effect at a behavioral level. Since the re-emergence of tDCS, great emphasis has been placed on conducting multi-modal investigations to establish the underlying principles of the technique (Venkatakrishnan and Sandrini, 2012; Hunter et al., 2013). Most recently, promising results from concurrent tDCS and Magnetoencephalography (MEG) interventions have been established (Soekadar et al., 2013), which could be used to derive crucial evidence of electrophysiological changes both during and after stimulation. Future research incorporating such measures of neuronal function could, therefore, be used to assess the presence of modulations in neurobiology that are not evident at the level of behavior.

## Author Contributions

CH: Experimental design, acquiring data, analyzing and inter preting results, writing initial manuscript, approving final manuscript.

MT: Assistance with implementation of tasks, providing access to prior data for power calculations, experimental design, theoretical background (vibrotactile tasks), editing manuscript, approving final manuscript.

DM: Experimental design, theoretical background (tDCS/vibrotactile tasks), interpretation of data, editing manuscript, approving final manuscript.

## Conflict of Interest Statement

The authors declare that the research was conducted in the absence of any commercial or financial relationships that could be construed as a potential conflict of interest.
