# Supplementary material for: Stimulating somatosensory psychophysics: a double-blind, sham-controlled study of the neurobiological mechanisms of tDCS
Source: Front Cell Neurosci. 2015 Oct 7;9:400. doi: 10.3389/fncel.2015.00400 (PMC4595660; doi:10.3389/fncel.2015.00400)
Supplement: Supplementary file 1 [file Data_Sheet_1.DOCX]

***Supplementary Material***

**Stimulating somatosensory psychophysics:
A double-blind, sham-controlled study of the neurobiological mechanisms of tDCS**

**Claire J. Hanley^1,^², Mark Tommerdahl³, and David J. McGonigle ^1,^²***

^1^Cardiff University Brain Research Imaging Centre, School of Psychology, Cardiff University, Cardiff, UK
^2^School of Biosciences, Cardiff University, Cardiff, UK
^3^Department of Biomedical Engineering, University of North Carolina, Chapel Hill, NC, USA

*** Correspondence:** David McGonigle, Cardiff University Brain Research Imaging Centre, School of Psychology, Cardiff University, Cardiff, CF10 3AT
McGonigleD@Cardiff.ac.uk

**Adverse Effects Questionnaire Items**

**Adverse Effects Questionnaire**

1.Sex: □ male

□ female

2. Age: ____

3. In how many studies did you participate? □ 1 study

□ 2-3 studies

□ 4-6 studies

□ more, approximately how many?_________

4. Where on the head were you stimulated (more than one possible if you participated in several studies)?

□ over the motor cortex (one electrode on the left side of the scalp and the other electrode over the right eye brow or vice versa)

□ over the visual cortex (one electrode on the back of the head and the other electrode over the centre of the head)

□ over the parietal cortex (one electrode behind the ear and the other electrode over the centre of the head)

□ over the frontal cortex (one electrode on the left forehead and the other electrode over the right eyebrow or vice versa)

5. Did you notice a flash either at the beginning or at the end of the experiment?

□beginning □end □neither

**During stimulation**

6. Did you experience any pain under the electrodes during stimulation

□ yes □ no

If yes – how strong was the pain?

1-marginal 2-moderate 3-middle-rate 4-strong 5-not tolerable

7. Was your scalp under the electrodes tingling during stimulation?

□ yes □no

If yes, how strong?

1-marginal 2-moderate 3-middle-rate 4-strong 5-not tolerable

8. Was your scalp itching underneath the electrodes during stimulation?

□ yes □no

If yes, how strong?

1-marginal 2-moderate 3-middle-rate 4-strong 5-not tolerable

9. Was your scalp burning underneath the electrodes during stimulation?

□ yes □no

If yes, how strong?

1-marginal 2-moderate 3-middle-rate 4-strong 5-not tolerable

10. Were you tired during stimulation?

□ yes □no

If yes, how tired were you?

1-slightly 2-moderately 3-middle-rate 4-heavily 5-extremely

11. Were you nervous during stimulation?

□ yes □no

If yes, how nervous were you?

1-slightly 2-moderately 3-middle-rate 4-heavily 5-extremely

12. Did you experience problems with concentration during stimulation?

□ yes □no

If yes, how strong were the concentration problems that you noticed?

1-marginal 2-moderate 3-middle-rate 4-strong 5-extreme

13. Did you experience problems of vision during the stimulation?

□ yes □no

If yes, how severe were the visual problems that you experienced?

1-marginal 2-moderate 3-middle-rate 4-strong 5-extreme

14. Did you suffer from a headache during the stimulation?

□ yes □no

15. Did you feel something unusual during the stimulation?

□ yes □no

If yes, please give a short description:_________________________________________

________________________________________________________________________

16. Did you feel anything else during stimulation?

□ yes □no

If yes, please give a short description:__________________________________________
________________________________________________________________________

17. Did you experience the stimulation as unpleasant?

□ yes □ no

If yes, how unpleasant?

1-slightly 2-moderately 3-middle-rate 4-heavily 5-extremely

**After stimulation**

18. Did you experience any pain underneath the electrodes after stimulation?

□ yes □ no

If yes, how strong was the pain?

1-marginal 2-moderate 3-middle-rate 4-strong 5-not tolerable

19. Was your scalp underneath the electrodes tingling after stimulation?

□ yes □no

If yes, how strong was the tingling?

1-marginal 2-moderate 3-middle-rate 4-strong 5-not tolerable

20. Was your scalp itching underneath the electrodes after stimulation?

□ yes □no

If yes, how strong was the itching?

1-marginal 2-moderate 3-middle-rate 4-strong 5-not tolerable

21. Was your scalp burning under the electrodes after stimulation?

□ yes □no

If yes, how strong was the burning?

1-marginal 2-moderate 3-middle-rate 4-strong 5-not tolerable

22. Were you tired after stimulation?

□ yes □no

If yes, how tired were you?

1-slightly 2-moderately 3-middle-rate 4-heavily 5-extremely

23.Were you nervous after stimulation?

□ yes □no

If yes, how nervous were you?

1-slightly 2-moderately 3-middle-rate 4-heavily 5-extremely

24. Did you experience problems with concentration after stimulation?

□ yes □no

If yes, how strong were the concentration problems?

1-marginal 2-moderate 3-middle-rate 4-strong 5-extreme

25. Did you experience any problems with vision after stimulation?

□ yes □no

If yes, how strong were the visual problems?

1-marginal 2-moderate 3-middle-rate 4-strong 5-extreme

26. Did you get a headache after stimulation?

□ yes □no

27. Did you feel sick after stimulation?

□ yes □no

If yes, how long did you feel sick (in hours)?___________________________________

28. Did you vomit after stimulation?

□ yes □no

If yes, how often?_______________

29.Did you experience any sleeping problems after stimulation?

□ yes □no

If yes, for how many days?_______________

30. Did you experience any mood changes after stimulation?

□ yes □no

If yes, for how long (in hours)?___________

31. Did you feel cold after stimulation?

□ yes □no

If yes, for how long (in hours)?___________

32. Did you feel warm after stimulation?

□ yes □no

If yes, for how long (in hours)?_____________

33. Did you experience anything unusual after stimulation?

□ yes □no

If yes, please give a short description:__________________________________________

________________________________________________________________________

34. Did you feel anything else after stimulation?
□ yes □no

If yes, please give a short description:__________________________________________

________________________________________________________________________

35. Did you realise any difference between different stimulation sites?

□ yes □no

If yes, please give a short description:__________________________________________

________________________________________________________________________

36. Were you anxious about the stimulation?

□ yes □no

37. Would you wish to participate again in a tDCS study?

□ yes □no

*Please check that you have answered all the questions.*

Thank you very much!

**Adverse Effects Questionnaire Data.**
Mean ratings on the severity of the adverse effect experienced during and after stimulation, on a scale of 1-5 (larger numbers indicate increased severity). Error bars represent ± 1 standard error (S.E.M).
